# Manufacturing and Characterization of Novel Electrospun Composite Comprising Polyurethane and Mustard Oil Scaffold with Enhanced Blood Compatibility

**DOI:** 10.3390/polym9050163

**Published:** 2017-05-04

**Authors:** Saravana Kumar Jaganathan, Mohan Prasath Mani, Ahmad Fauzi Ismail, Manikandan Ayyar

**Affiliations:** 1 Department for Management of Science and Technology Development, Ton Duc Thang University, Ho Chi Minh City, Vietnam; 2 Faculty of Applied Sciences, Ton Duc Thang University, Ho Chi Minh City, Vietnam; 3 IJN-UTM Cardiovascular Engineering Centre, Faculty of Biosciences and Medical Engineering, Universiti Teknologi Malaysia, Skudai 81300, Johor, Malaysia; 4 Faculty of Biosciences and Medical Engineering, Universiti Teknologi Malaysia, Skudai 81300, Johor, Malaysia; mohanprasathutm@gmail.com; 5 Advanced Membrane Technology Research Centre (AMTEC), Universiti Teknologi Malaysia, Skudai 81310, Johor, Malaysia; afauzi@utm.my; 6 Department of Chemistry, Bharath University, Chennai, Tamil Nadu 600073, India; manikandana.che@bharathuniv.ac.in

**Keywords:** polyurethane, mustard oil, composite, cardiac patches, electrospun

## Abstract

The objective of this work is to characterize and investigate the blood compatibility of polyurethane (PU)/mustard oil composites fabricated using electrospinning technique. The fabricated scaffold was characterized using scanning electron microscopy (SEM), Fourier transform infrared spectroscopy (FTIR), atomic force microscopy (AFM), thermogravimetric analysis (TGA) and contact angle measurements. The activated partial thromboplastin time (APPT), prothrombin time (PT) and the hemolytic assay were done to investigate the blood compatibility of the developed composites. The SEM results revealed that the fiber diameter of the composites (761 ± 123 nm) was reduced compared to pristine PU control. The interaction between PU and mustard oil was confirmed by FTIR as evident through the shifting of peaks. The fabricated composites depicted hydrophobic behavior as insinuated by the increase in contact angle measurements. PU/mustard composites displayed improved crystallinity as confirmed by TGA. Atomic force micrographs suggested that developed PU/mustard oil composites showed an increase in the surface roughness (*R*a) compared to pure PU. The Ra of pure PU was observed to be 723 nm but for the fabricated PU/mustard oil composite the *R*a was found to be 1298 nm (*R*a). The hemolytic index value for pure PU and fabricated composites was observed to be 2.73% and 1.15% indicating that developed composites showed a non-hemolytic behavior signifying the safety of the composites with red blood cells. Hence the newly developed composites with improved physicochemical and blood compatibility properties may be considered as a potential candidate for fabricating cardiac patches and grafts.

## 1. Introduction

Cardiovascular disease (CVD) has become a leading global threat of the 21st century and is considered as one of the most common causes of death in the developed countries. The main cause for heart failure is the myocardial infarction (MI) which results in impairment of cardiac tissue and loss of left ventricular function [1]. The impairment of the heart wall muscle is permanent because the myocardial tissue lacks the significant intrinsic regenerative capability to replace the lost cells. Therefore, there is a need for standard clinical procedure to restore the damaged myocardium other than heart transplantation. Due to existing limitations, researchers are forced to identify novel and more feasible alternatives ways to develop a cardiac construct for treatment and regeneration of the heart. Cardiac tissue engineering (TE) is an emerging method and considered as an alternate method to repair the damaged cardiac tissue with the aid of cellular transplantation and biomaterial scaffolds [2]. Several research works have been carried out in vitro and in vivo and the fabricated scaffolds show great potential towards the remodeling of damaged cardiac tissue. However, the fabricated scaffolds should possess essential qualities like thromboresistant and anticoagulant nature in order to prevent the graft failure [3]. The fabricated patch comes in direct contact with red blood cells and it should not induce any damage to the red blood cells. Thromboresistant properties are assessed by the measurement of activated partial thromboplastin time (APPT) and prothrombin time (PT) [4]. These two time points are good indicators of intrinsic and extrinsic pathways, respectively. The hemolytic percentage may serve as a yardstick for estimating the damage to red blood cells.

Fabrication of fiber based scaffold in cardiac tissue engineering with enhanced extracellular matrix (ECM) was still challenging in clinical applications for cell adhesion, assembly, and growth. The electrospun fibrous scaffolds possess many advantages like the high surface area to volume ratios, controlled fiber diameters and the capacity to add multiple polymers and bioactive ingredients for attaining suitable mechanical and biodegradation properties. Owing to the above characteristics, the electrospun nanofibers are widely used in tissue engineering (TE) [5,6,7,8]. Electrospinning is a method used to producing fibers with average diameters in the nanometer range. Electrospinning as a technique in the medical field had received the attention in the recent decade for producing nonwoven nanofiber meshes in making scaffolds with controlled microstructure. In electrospinning, the parameters like properties of the native polymer, the rheological properties of its solution, the voltage applied and the diameter of the orifice significantly influenced the polymer solution jet during the fabrication of nanofibers [9,10,11]. In this research, the polymer used to fabricate nanofibers was tecoflex EG80A which is a poly-ether based medical grade polyurethane. Polyurethane (PU) polymer was used in making nanofibers because it possesses good barrier properties and oxygen permeability which allows cells to proliferate more and makes them suitable for tissue regeneration [9].

The reinforcement used was mustard oil which occurs naturally in a wide range of the seeds, stems, leaves, and roots of cruciferous plants such as mustard, broccoli, horseradish, cabbage, cauliflower, kale and turnips. The mustard oil mainly contains allyl isothiocyanate (AIT) which is a non-phenolic volatile compound that found in crucifereae plants [12]. Mustard oil is classified into two different varieties with the seeds as their major source. The first type is directly extracted as a fatty vegetable oil obtained by crushing the seeds whereas the second one is manufactured by grinding the seeds and allowed to mix it with water and thereby collect the volatile oil by the process of distillation. Mustard oils were used to control the food spoilage and food borne pathogenic bacteria [13,14]. In this study, PU and mustard oil composites will be fabricated using one-step electrospinning and investigated for its physicochemical properties and blood compatibility.

## 2. Materials and Methods

The medical grade tecoflex EG-80A thermoplastic PU polymer was purchased from Lubrizol, Wickliffe, OH, USA. The solvent for polyurethane was *N*,*N*-dimethylformamide (DMF) supplied by Merck Millipore, Darmstadt, Germany. The commercially available mustard oil (Renga′s trading, Johor, Malaysia) was obtained locally. The chemicals phosphate buffered saline (PBS) and sodium chloride physiological saline (0.9% *w*/*v*) was obtained from Sigma-Aldrich, Kuala Lumpur, Malaysia. The reagents utilized in APTT and PT assay such as rabbit brain activated cephaloplastin, calcium chloride (0.025 M), and thromboplastin (Factor III) were supplied by Diagnostic Enterprises, Solan, India.

### 2.1. Preparation of Composite

The homogeneous solution of the polymer with a concentration of 8% (*w*/*v*) was prepared by dissolving 480 mg of PU in6 ml of DMF by magnetic stirring for 24 h at room temperature. Similarly, the homogeneous solution of mustard oil was done by mixing 400 μL of mustard oil with 4.6 mL of DMF to make 8% *v*/*v* solution and stirred for 1 h minimum at room temperature. Finally, the prepared homogeneous solution of PU and mustard oil was mixed slowly at a ratio of 8:2 (*v*/*v*) to fabricate PU/mustard oil composite.

### 2.2. Fabrication of PU and Composite Scaffold

The electrospinning technique was utilized to fabricate pure PU nanofiber and PU/mustard oil composite. Initially, the prepared solutions of PU and composite was drawn into the plastic syringe of 10 mL of 18-G stainless steel needle and attached to the syringe pump (SP20, NFiber). To fabricate electrospun nanofibers a supply voltage was given by the NFiber high voltage unit. The grounded static collector drum was used to collect the developed nanofibers covered with aluminum foil. In our study, the nanofibers were fabricated at a flow rate of 1.0 mL/h with an applied voltage of 10 kV. Since the PU solution containing mustard oil had reduced viscosity, the flow rate and voltage were changed to 0.50 mL/h and 7 kV respectively, to acquire a steady jet of the polymer solution. For both samples, the collector distance was placed at 16 cm and maintained constant. The deposited nanofibrous mesh on the collector drum was carefully isolated and dried at room temperature for 24 h.

### 2.3. Physicochemical Characterization

#### 2.3.1. Scanning Electron Microscopy (SEM) Micrographs

The SEM unit (Hitachi Tabletop TM3000, Tokyo, Japan) was used to analyze the surface characteristics of the electrospun PU and the mustard oil composite. Before SEM analysis the samples were gold coated to attain photomicrographs. Then, Image J (National Institutes of Health, Bethesda, MD, USA) software was used to calculate the fiber diameter size distribution in the developed membranes by measuring at least 30 individual fibers randomly. The mean and standard deviation of the diameter distribution was obtained from Image J software. The thickness of electrospun PU and the mustard oil composite was found to be 0.45 ± 0.03 and 0.47 ± 0.01 mm respectively.

#### 2.3.2. Attenuated Total Reflectance Fourier Transform Infrared Spectroscopy (ATR-FTIR) Analysis

The ATR-FTIR unit (Nicolet is 5, Thermo Fischer Scientific, Waltham, MA, USA) was used to investigate the chemical composition of the electrospun PU and the mustard oil composite. A small amount of the sample was placed on the sensor surface and the spectra were inspected to investigate the chemical compositions. In the interim, the IR spectra of mustard oil were obtained by placing a drop of the sensor surface. The spectra of each sample were measured over the range of 600–4000 cm^−1^ at 32 scans per minute and averaged at the resolution of 4 cm^−1^. Zinc Selenium (ZnSe) was used as an ATR crystal which was fixed with the NICOLET IS5 spectrometer. The spectra were baseline corrected and normalized using Spekwin 32 software (Society for Applied Spectroscopy, Berchtesgaden, Germany) to inspect the active group and peaks that present in the fabricated membranes. 

#### 2.3.3. Contact Angle Measurements 

The VCA Optima contact angle measurement unit (AST Products, Inc., Billerica, MA, USA) was used to analyze the wettability of electrospun PU and the mustard oil composite. Sample with a size of 1 × 5 cm^2^ was cut from the fabricated mesh and placed on the surface for calculating the contact angle. For performing contact angle measurements, the setup of the syringe filled with the water was done and then a droplet of size 2 μL was formed at the tip and it was made to fall on the test membrane. Using a high resolution camera, the image of the static liquid deposition was obtained within few seconds. The experiment was repeated for three different trails and with the support of computer integrated software, the manual contact angle was obtained.

#### 2.3.4. Thermogravimetric Analysis

The TGA unit (PerkinElmer, Waltham, MA, USA) was used to perform the TGA analysis of electrospun PU and the mustard oil composite. The experiment was performed under a dry nitrogen atmosphere in the temperature range 30–1500 °C at an ascending rate of 10 °C/min by placing a small piece of sample weighing 3 mg in an aluminum pan. At each temperature point, the remaining weight residue was recorded and the obtained values were exported in an excel sheet. Then using Origin Pro 8.5 software the TGA curve and the corresponding derivative weight loss curve (DTG) were drawn.

#### 2.3.5. Atomic Force Microscopy

To investigate the surface morphology of the sample the atomic force microscopy (NanoWizard^®^, JPK Instruments, Berlin, Germany) was used and the machine integrated JPJSPM software was used to attain a 3D image of the sample surface. The sample with a size of 1 × 1 cm^2^ was cut from the fabricated membrane to evaluate the surface roughness of fabricated samples and placed on the AFM equipment (Nanowizard, JPK instruments, Berlin, Germany). The AFM analysis was performed in the normal atmosphere at room temperature in the event of measuring surface roughness. The images with the scanning size of 20 μm × 20 μm were captured and recorded in the medium mode with 256 × 256 pixels. Three measurements were carried at various positions to measure the average surface roughness (*R*a). 

### 2.4. Hemocompatibility Assessment of the Scaffold Material

#### 2.4.1. Ethical Statement and Collection of Blood Samples

All the experimental procedures incorporated in the treatment of blood were approved by the Faculty of Biosciences and Medical Engineering, Universiti Teknologi Malaysia with ref no: UTM.J.45.01/25.10/3Jld.2(3). The blood was collected from healthy adults who were educated about the risk and benefits of the blood donation. The blood was collected after getting a signature of consent via venipuncture. The collected blood was anticoagulated with acid-citrate-dextrose (ACD) (56 mM sodium citrate, 65 mM citric acid, 104 mM dextrose) at a ratio of 9:1 (blood/citrate). Finally, the platelet poor plasma (PPP) extract was obtained by centrifuging the citrated blood at 3000 rpm for 15 min respectively. 

#### 2.4.2. Activated Partial Thromboplastin Time (APTT) Assay 

Fabricated PU and the PU/mustard oil scaffold were cut into square samples of dimension 0.5 × 0.5 cm^2^. The assay was carried out in triplicate, so three square samples were gently washed with deionised water and introduced into 96-well plates. The samples were soaked in phosphate-buffered saline (PBS) bath and incubated at 37 °C for 30 min before starting the assay. Then 50 μL of the obtained platelet-poor plasma was introduced on the 96-well plates in which the sample placed and incubated for 1 min at 37 °C. After this, 50 μL of rabbit brain cephaloplastin reagent was mixed and incubated for 3 min at 37 °C followed by addition of 50 μL of calcium chloride (CaCl_2_). The mixture was stirred using the needle and the blood clot time was measured using chronometer. 

#### 2.4.3. Prothrombin Time (PT) Assay

For PT assay, the fabricated nanofibrous membrane was cut into square samples as described in the “APTT assay” section, and the test was also performed in triplicate. The samples were washed with deionized water and incubated in PBS for 30 min at 37 °C. It was further incubated in 50 μL of platelet-poor plasma at 37 °C for 1 min followed by 50 μL of sodium chloride (NaCl)–thromboplastin reagent (Factor III). The mixture was stirred using the needle and the time required until the blood clot was measured using a chronometer. 

#### 2.4.4. Hemolysis Assay

The hemolysis assay was performed to determine the effect of fabricated membranes on red blood cells (RBC’s) using citrated whole blood. Firstly, both PU and bio-nanofibrous samples (1 × 1 cm^2^) were incubated in physiological saline (0.9% *w*/*v*) bath at 37 °C for 30 min. Afterwards, the resulting sample was incubated with a mixture of aliquots of citrated blood and diluted saline at a ratio of 4:5 (*v*/*v* %) for 1 h at 37 °C. To prepare positive and negative controls the whole blood was diluted with distilled water (4:5) for complete hemolysis along with physiological saline solution respectively. After incubation, the mixture was centrifuged at 3000 rpm for 15 min and the release of hemoglobin was determined by photometric analysis of the supernatant at 545 nm which indicates the RBC damage. Finally, the percentage of hemolysis or hemolytic index was calculated using the formula [15].

Hemolysis ratio (HR) = (TS − NC)/(PC − NC) × 100

where TS, NC, and PC are measured absorbance values of the test sample, negative control and positive control at 542 nm, respectively.

#### 2.4.5. Statistical Analysis

All experiments were conducted thrice independently. Unpaired *t*-test was done to determine statistical significance. The results obtained from all experiments are expressed as mean ± SD. In the case of qualitative experiments, a representative of three images is shown.

## 3. Results and Discussion

**Figure 1:**
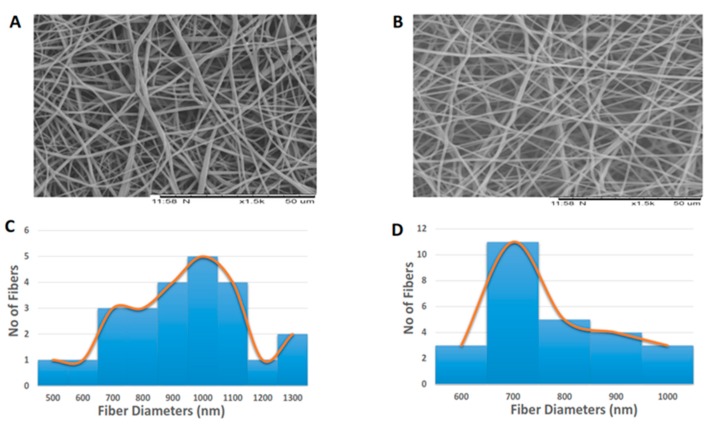
SEM images of (**A**) Polyurethane (**B**) Polyurethane/mustard oil composites (**C**) Fiber diameter of polyurethane (PU) (**D**) Fiber diameter of PU/mustard oil composites.

Figure 1A,B indicates the SEM image of prepared polyurethane/mustard oil composites and pure PU. The surface morphology of fabricated composites showed the fine dispersion of nanofibers with randomly oriented in the matrix. The morphology study showed that the polymer PU has fibers with mean diameter and standard deviation of 916 nm ± 216 nm and for developed composites, the fiber diameter was found to be 761 ± 123 nm respectively. The statistical analysis of those fiber diameters indicated significant differences (*p* < 0.05) in their mean value. The fiber diameter distribution curves of pure PU and fabricated PU/mustard oil composites were shown in Figure 1C,D. It was observed that the prepared PU/mustard oil composites showed reduced fiber diameter compared to pure PU. Balaji et al. prepared scaffold based on polyurethane added with honey and papaya and investigated the hemocompatibility of developed scaffold. It was observed that the developed scaffold showed decreased fiber diameter and our obtained results were observed to be similar to their observations [15]. The fiber diameter of 600–1200 nm resulted in the highest proliferation of fibroblast cells compared with other diametric ranges as reported by Kumbhar et al. and also they observed within these ranges of diameters an increased collagen III expression [16]. Hence, it is surmised that our fabricated scaffolds may be beneficial in promoting the fibroblast cell growth. Mengyan et al. developed poly (lactic-*co*-glycolic acid, PLGA) scaffold blended with gelatine and elastin components for soft tissue engineering. The fabricated PLGA-gelatine-elastin fibers were homogeneous and also found to be reduced diameter compared to pure PLGA. It was found that the developed membrane was more compatible to H9C2 rat cardiac myoblast cells attachment and proliferation which favors the tissue growth [17]. Our composites displayed a similar reduction pattern in the diameter which may be compatible with the growth of cardiac cells.

**Figure 2:**
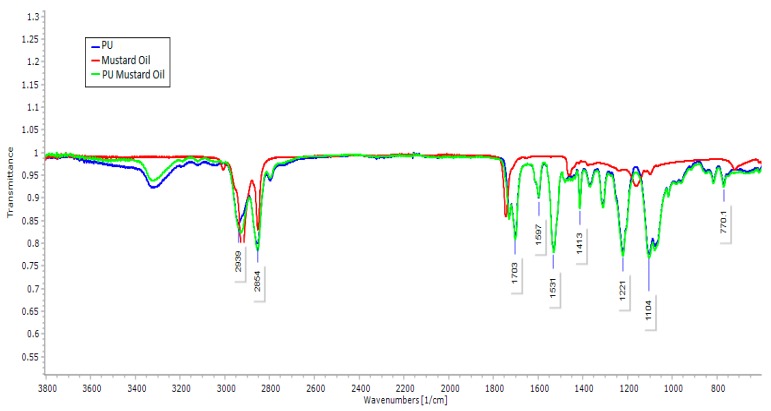
Fourier transform infrared spectrum (FTIR) of PU, mustard oil and PU/mustard oil composites.

The results of FTIR analysis of prepared samples, mustard oil and pure PU are represented in Figure 2. FTIR spectra of PU samples showed a broad peak at 3323 cm^−1^, which indicates the presence of hydroxyl (NH) stretching of aliphatic primary amine and the NH vibrations were indicated by the peak 1597 and 1531 cm^−1^. The bands at 2939 and 2853 cm^−1^ were attributed to carbonyl (CH) stretching and the peak at 1413 cm^−1^ represents the presence of vibrations of CH stretching respectively. The characteristic band at 1730 and 1703 cm^−1^ was identified as a twin peak indicating the presence of carboxylic (C=O) stretching. The presence of sharp peaks at 1221, 1104, 770 cm^−1^ denotes the C–O stretching corresponding to alcohol groups [18,19]. Similarly, in mustard oil infrared (IR) spectrum, the results showed a sharp absorption band frequency at 2922 cm^−1^ and 2852 cm^−1^ indicating the CH stretching and the other characteristic band at 1744 cm^−1^ was attributed to C–O stretching respectively. The vibrations of CH stretching and C–O corresponding to alcohol was denoted by the characteristic band of 1463 cm^−1^ and 1175 cm^−1^ respectively. From IR spectrum of PU/mustard oil composites, the results clearly showed that the absorption bands of PU were found to be decreased with the formation of hydrogen bonding [20]. The evidence for the interaction between PU and mustard oil was showed by a slight shifting of CH stretching band at 2939 cm^−1^ in pure PU to 2929 cm^−1^ in PU/mustard oil mats and concluded the presence of mustard oil in the polyurethane matrix [21].

The results of contact angle measurements of PU/mustard oil composites and pure PU was shown in Table 1. In our study, the contact angle measurement of PU/mustard oil sample was found to be 117° and for pure PU it was found to be 86° which clearly proves that the addition of mustard-oil into the polymer matrix induced the hydrophobicity in the prepared samples. It has been reported by Ceylan et al. and Cui et al. that the smaller fiber diameter could also result in increase in water contact angle which was observed in this work [22,23]. Yin et al. developed electrospun vascular graft based on collagen/chitosan/poly(l-lactic acid-*co*-ɛ-caprolactone), P(LLA-CL). In this study, the contact angle of P(LLA-CL) was found to be 136.1 ± 1.3 and for the P(LLA-CL) blended collagen and chitosan scaffolds were found to be 110.5 ± 0.9. Moreover, it was observed that the P(LLA-CL) blended collagen and chitosan scaffolds showed enhanced endothelial cell adhesion and cell proliferation compared to pure P(LLA-CL) [24]. In another study, Xiang et al. developed an electrospun tubular scaffold using pNSR32 (recombinant spider silk protein), polycaprolactone (PCL) and gelatin. It was observed that the contact angle of PCL was found to be 109.9 ± 8.6 while for PCL blended pNSR32 scaffold displayed an increased value of 114.3 ± 5.9. Moreover, the PCL blended pNSR32 scaffold showed more cell viability and proliferation compared to pure PCL [25]. In our study, the contact angle of fabricated composites was observed to be within these reported values and it might be suitable for the cardiovascular cellular growth.

**Figure 3:**
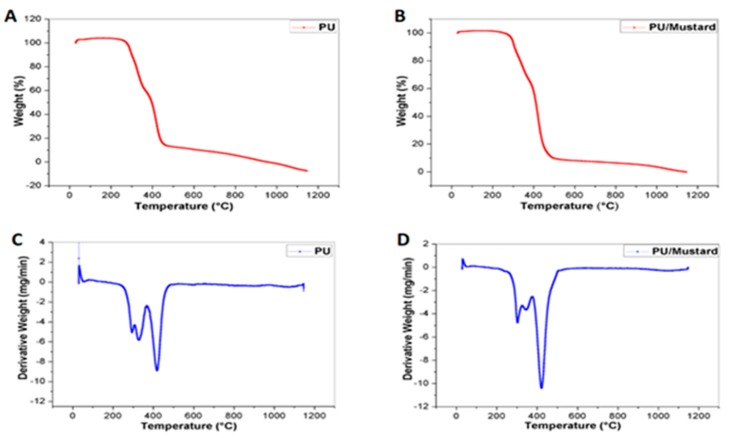
Thermogravimetric analysis (TGA) analysis of (**A**) Pure Polyurethane (**B**) Polyurethane/mustard oil composites (**C**) Weight residue percentage of Pure PU and (**D**) Weight residue percentage of PU/mustard oil composites.

The thermal degradation behavior of prepared composites and pure PU was calculated using TGA analysis. The thermal analysis was used to analyze the integrity and also durability of the prepared composites [26] and the electrical properties have a role in determining the cell adhesion and proliferation [27]. The results obtained from TGA showed that the onset degradation temperature of PU and PU/mustard oil composites was found to be 273 and 290 °C respectively, as shown in Figure 3A,B. The highest degradation behavior pattern of PU/mustard oil may be due to incorporated constituents of mustard oil in the PU nanofibers. Further, in this study, we calculated the percentage residual weight loss of PU and the prepared composites at 900 °C which was found to be 1.91% and 5.36% as shown in Figure 3C,D which clearly indicates the significant improvement in thermal stability of the prepared composite samples. Yan et al. studied the thermal and electrical properties of polystyrene-graphene nanofibers via electrospinning technique. It was found that the addition of graphene nanoparticles into the polymer matrix increased the thermal stability of polystyrene and our analyzed results were observed to be similar to their findings [28].

**Figure 4:**
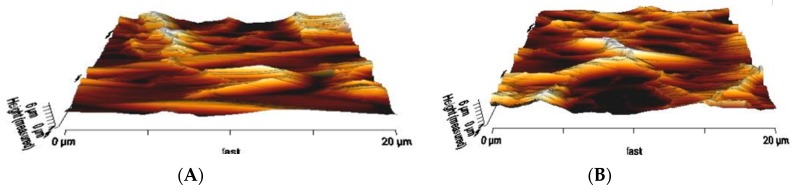
Atomic force microscopy (AFM) analysis of (**A**) Polyurethane and (**B**) Polyurethane/mustard oil composites.

The surface roughness of the PU surface and PU/mustard oil composites that derived from AFM was shown in Figure 4A,B. Atomic force micrographs suggested that developed PU/mustard oil composites showed an increase in the surface roughness (*R*a) compared to pure PU. The Ra of pure PU was observed to be 723 nm but for the fabricated PU/mustard oil composite, the Ra was found to be 1298 nm (*R*a). The inferred surface roughness was due to the presence of mustard oil constituents in the polymer matrix. Parvinzadeh et al. studied the surface properties of polyethylene terephthalate (PET)/clay composites and observed that the addition of clay into PET improved the surface roughness. It was reported that the increase in surface roughness was due to changes in the particle-particle and particle-PET chain interactions [29]. The successful blending of mustard oil into the pure PU matrix resulted in the interactions between the mustard oil particles and PU chain which may result in the enhanced surface roughness. 

**Figure 5:**
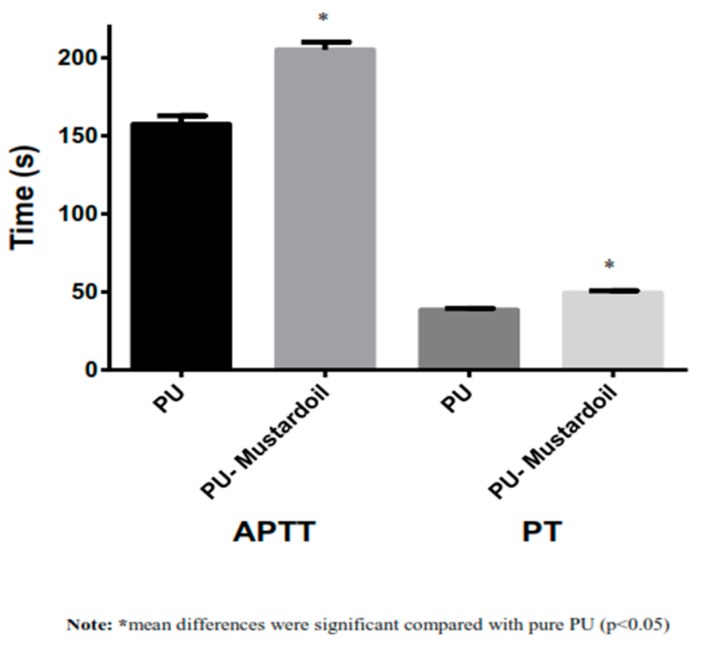
Activated Partial Thromboplastin Time (APTT) and prothrombin time (PT) assay of Polyurethane and polyurethane/mustard oil composites.

**Figure 6:**
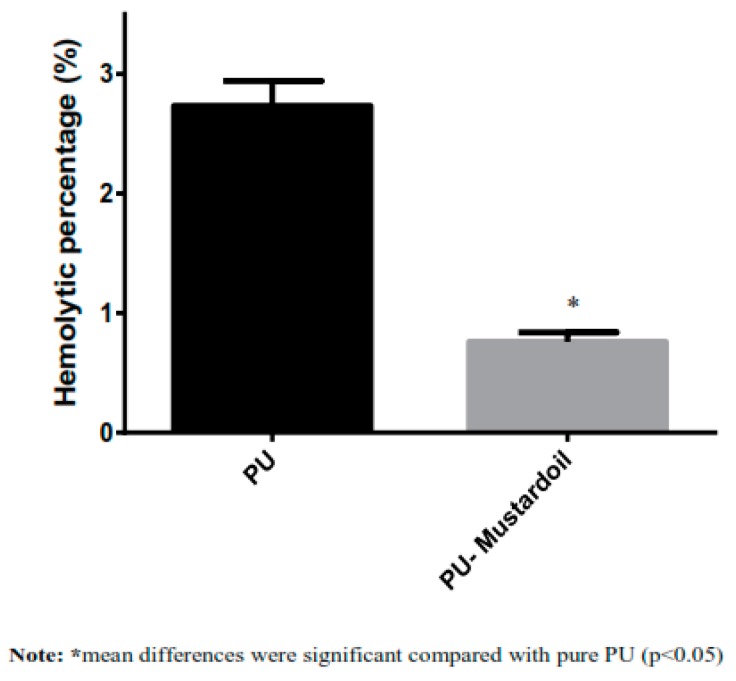
Hemolysis assay of Polyurethane and polyurethane/mustard oil composites.

Blood clotting and anticoagulant properties of pure PU and PU/mustard oil were investigated using the APT and PT assay. To investigate the extrinsic coagulation pathway, the PT assay was used, and for intrinsic coagulation pathway, the APTT was used. The APTT values of developed hybrid PU/mustard oil composites showed prolonged blood clotting time of about 205 ± 5 s compared to pure PU membrane where the blood clotting time was found to be about 157 ± 5.55 s, as indicated in Figure 5. The increase in values of blood clotting time clearly shows that the composite has better surface compared to pure PU and also enhances the anticoagulant nature of PU composite by delaying the intrinsic pathway. Also, in the PT measurement assay, the PU/mustard oil composites showed enhanced blood clotting time of about 49 ± 2 s while that for pure PU was observed to be 38 ± 1 s, as indicated in Figure 5. The increase in blood clotting time of PU/mustard oil composites was due to several factors. Increased surface roughness may be one of the contributors, as observed in the AFM analysis. Milleret et al. optimized fiber diameter in developing scaffolds by using two types of polymers, namely degarapol and poly (lactic-*co*-glycolic acid) (PLGA), and reported that smaller fiber diameter showed a delay in blood clotting. The research paper also concluded that the smaller fiber diameter will be conducive to blood compatibility which seems to be similar to the observed findings [30]. Moreover, the delay in blood clotting may be due to the addition of mustard oil constituents into PU matrix. Hemolysis assay is a very simple and more reliable way to analyze the safety of the hybrid fabricated patch against red blood cells. The hemolytic percentage was computed by recording the absorbance of obtained supernatant after blood reacts with the composite patch at 542 nm. From the measurement of the hemolytic assay, it was found that the absorbance value of PU was found to be high compared to PU/mustard oil composites, indicating lysis of erythrocytes by pristine PU. According to ASTMF756-00(2000) standard, it was concluded that the smaller the hemolysis value, the better would be the safety of red blood cells. In agreement with the above standard, it has been described that when the hemolytic value is above 2%, the material is considered to be hemolytic and if the index value less than 2% then it was considered to be non-hemolytic [31]. The hemolytic percentage of fabricated composite material was found to be 0.76% while for pure PU the index was observed to be 2.73%, as shown in Figure 6. Hence, in our fabricated patches, the hemolytic index is within the permissible limits which were below 1% and it could be considered as a highly non-hemolytic material. Ruiming et al. investigated the blood compatibility of the bio-inspired small vascular graft using polyurethane. It was found that the topography was observed to be hydrophobic with the contact angle of 132° ± 3.5°, possessing better blood compatibility [32]. Our fabricated composites were hydrophobic with better blood compatibility, which may be suitable for fabricating small vascular grafts.

## 4. Conclusions

In this work, a scaffold based on PU and mustard oil was successfully prepared by a one step electrospinning technique. It was observed that the developed composites had smaller fiber diameter in the range of less than 1000 nm. The FTIR spectrums showed an interaction between PU and mustard oil by the formation of a hydrogen bond. The experimental results of contact angle have shown that composites rendered the hydrophobic surface and also exhibited higher thermal stability due to the existence of mustard oil constituents. The introduction of mustard oil into polymer matrix has favored the change in surface morphology with enhanced surface roughness, as evident by AFM analysis. The APTT and PT assay revealed the delay in blood clotting time of the fabricated composites. Further, the hemolytic index value of PU/mustard oil composites were observed to very low compared to pristine control, indicating the enhanced safety of the fabricated material with RBC. Hence, the newly developed nanofibrous membrane based on PU/mustard oil components with enhanced physicochemical structure and blood compatibility might be used as the alternative to the substrate scaffold for damaged blood tissue vessels.

## Figures and Tables

**Table 1:** Contact angle measurements of Polyurethane and Polyurethane/mustard oil composites.

S. No	Sample	Average contact angle in degrees
1	Pure Polyurethane	86 ± 1.91
2	Polyurethane/mustard oil composites	117 ± 1.83 *

Note: * mean differences were significant compared with pure PU (*p* < 0.05).
